# Method parameters’ impact on mortality and variability in rat stroke experiments: a meta-analysis

**DOI:** 10.1186/1471-2202-14-41

**Published:** 2013-04-01

**Authors:** Jakob O Ström, Edvin Ingberg, Annette Theodorsson, Elvar Theodorsson

**Affiliations:** 1 Department of Clinical and Experimental Medicine, Clinical Chemistry, Faculty of Health Sciences, Linköping University, County Council of Östergötland, Linköping, Sweden; 2 Department of Clinical and Experimental Medicine, Neurosurgery, Faculty of Health Sciences, Linköping University, County Council of Östergötland, Linköping, Sweden

**Keywords:** Brain infarction, Middle cerebral artery occlusion, Rats, Methods, Mortality, Variability

## Abstract

**Background:**

Even though more than 600 stroke treatments have been shown effective in preclinical studies, clinically proven treatment alternatives for cerebral infarction remain scarce. Amongst the reasons for the discrepancy may be methodological shortcomings, such as high mortality and outcome variability, in the preclinical studies. A common approach in animal stroke experiments is that A) focal cerebral ischemia is inflicted, B) some type of treatment is administered and C) the infarct sizes are assessed. However, within this paradigm, the researcher has to make numerous methodological decisions, including choosing rat strain and type of surgical procedure. Even though a few studies have attempted to address the questions experimentally, a lack of consensus regarding the optimal methodology remains.

**Methods:**

We therefore meta-analyzed data from 502 control groups described in 346 articles to find out how rat strain, procedure for causing focal cerebral ischemia and the type of filament coating affected mortality and infarct size variability.

**Results:**

The Wistar strain and intraluminal filament procedure using a silicone coated filament was found optimal in lowering infarct size variability. The direct and endothelin methods rendered lower mortality rate, whereas the embolus method increased it compared to the filament method.

**Conclusions:**

The current article provides means for researchers to adjust their middle cerebral artery occlusion (MCAo) protocols to minimize infarct size variability and mortality.

## Background

Ischemic stroke is amongst the leading causes of death and disability in the world and has been the subject of massive research efforts during recent years. Even though these efforts have resulted in more than 600 treatments reported effective in preclinical studies [1], clinically proven treatment options are still few. There are reasons to believe that this apparent translational roadblock may inter-alia be due to methodological confounding factors, including high mortality and large outcome variability, in the preclinical studies [2-4].

The usual approach in experimental stroke studies, used in hundreds of publications each year, is that A) focal cerebral ischemia is inflicted in rodents [5,6], B) some type of treatment is administered and C) the infarct sizes are assessed. Even though this setup may seem straight forward, there are infinite numbers of methodological variants, and there is a profound lack of consensus regarding the ideal methodology to be used in experiments of this kind. A small number of studies aiming to optimize the infarct induction regarding the important aspects of mortality and variability, for example by testing various rodent strains and sizes [7-9], surgical procedures [10-13] or occluding intraluminal filaments [14], have been published. However, these studies have rendered conflicting results, and are too few and too small to provide comprehensive understanding of how the different methodological parameters interact.

Hence, it was thought of interest to investigate the influence of different methodological factors on infarct variability and mortality in rat stroke models using a hypothesis-driven meta-analytical approach where their interactions and complexity could be embraced rather than disregarded. The meta-analytical approach seemed attractive since such a vast number of studies with the abovementioned experimental setup are published, and since control/vehicle/placebo groups (hereby referred to as “control groups”), suitable for inter-study comparisons, are almost invariably included. A study of this kind has, to the best of our knowledge, not been published previously. Even though other animals, not least mice, are also well-used in experimental stroke research, rats were due to space restrictions chosen to be the sole focus of the current article.

The aim of the current meta-analysis was to investigate chosen methodological variables’ effects on infarct size variability and mortality. An a priori hypothesis of six main factor-outcome relations (1A-3B) was established:

1. Rat strain affects (A) infarct size variability and (B) mortality.

2. Type of focal ischemia procedure affects (A) infarct size variability and (B) mortality.

3. In studies using the intraluminal filament method, the type of filament affects (A) infarct size variability and (B) mortality.

## Methods

### Article inclusion

To identify articles to be included in the meta-analysis, Medline was searched with the line *(mcao or “middle cerebral artery occlusion” or “MCA occlusion” or “stroke” or “cerebral ischemia” or “brain ischemia”) and (rat or rats)*, resulting in more than 19,000 hits. Starting with the latest article the 10^th^ of June 2011 [15], the articles were consecutively, in order of PubMed identifier, assessed for inclusion in the study.

The inclusion criteria were:

A. Article written in English

B. Original research article

C. Experiments performed in living adolescent, adult or elderly rats

D. Animals inflicted one single focal cerebral ischemic lesion

E. Infarct size assessed and results presented

F. Inclusion of a control group, untreated except for vehicle treatments

G. Sufficient description of fundamental aspects of the experiment (after e-mail correspondence)

### Data extraction

Data about the control groups were extracted from all included articles. If an article included more than one control group, differing in for example euthanasia time-point, all control groups were separately included and assessed independently of each other. When extracting the method data, we adhered strictly to the principle “If it is not described, it was not performed”. Registered factors and outcome measures are listed in Table 1.

**Table 1:** Extracted factors and outcome measures

**Factor/Outcome measure**	**Data type**	Final categories* or unit	**Reference category for regression analyses**
**Rat property factors**			
*Strain*	Category	I. *Sprague Dawley*	*Sprague Dawley*
		II. *Wistar*	
		III. *SHR*	
		IV. *WKY*	
		V. *Other strains*	
*Sex*	Category	I. *Male*	*Male*
		II. *Female*	
		III. *Ovx female*	
		IV. *Mix or not specified*	
Elderly rats **	Category, Binomial	[No]	[No]
		[Yes]	
*Weight*	Continuous	Grams	NA
*Animal exclusion rate*	Continuous	%	NA
**Anesthesia factors**			
*Type of anesthetic*	Category	I. *Inhalation anesthetic*	*Inhalation anesthetic*
		II. *Chloral hydrate*	
		III. *Ketamine*	
		IV. *Barbiturates and benzodiazepines*	
		V. *Other anesthetics*	
*Intubation*	Category, Binomial	[No]	[No]
		[Yes]	
*Awakening of rats during occlusion*	Category, Binomial	[No]	[No]
		[Yes]	
*Temperature feedback system*	Category, Binomial	[No]	[No]
		[Yes]	
*Electroencephalographic surveillance*	Category, Binomial	[No]	[No]
		[Yes]	
*Blood pressure monitored*	Category, Binomial	[No]	[No]
		[Yes]	
*Heart rate monitored*	Category, Binomial	[No]	[No]
		[Yes]	
*Blood gases/O*_*2*_*saturation analyzed*	Category, Binomial	[No]	[No]
		[Yes]	
*Blood hemoglobin concentration analyzed*	Category, Binomial	[No]	[No]
		[Yes]	
*Blood glucose concentration analyzed*	Category, Binomial	[No]	[No]
		[Yes]	
*Postoperative antibiotics*	Category, Binomial	[No]	[No]
		[Yes]	
**Focal ischemia procedure factors**			
*Type of middle cerebral artery occlusion procedure*	Category	I. *Intraluminal filament*	*Intraluminal filament*
		II. Direct, mechanical***	
		III. *Embolic*	
		IV. *Photothrombotic*	
		V. *Endothelin injection*	
Occlusion duration****	Category	I. *Short transient (up to 60 minutes)*	*Short transient (up to 60 minutes)*
		II. *Long transient (>60 min)*	
		III. *Permanent*	
*Occluding filament type (Only studies using the intraluminal filament method)*	Category	I. *Uncoated*	*Uncoated*
		II. *Silicon coated*	
		III. *Poly-L-Lysine coated*	
		IV. *Other coatings*	
**Analysis procedure factors**			
*Time after focal ischemia for evaluation of damage*	Continuous	Hours	NA
*Type of infarct size evaluation*	Category	I. *TTC*	*TTC*
		II. *Radiology*	
		III. *Acidic/Basic stain or silver stain histology*	
		IV. *Immunohistology*	
*Edema correction used*	Category, Binomial	[No]	[No]
		[Yes]	
*Blinding of infarct size determination procedure*	Category, Binomial	[No]	[No]
		[Yes]	
*Criteria for excluding rats*	Category	I. *None*	*None*
		II. *Observed absence of cerebral blood flow reduction*	
		III. *Lack of functional deficit*	
		IV. *Too small infarct*	
		V. *Other pathology in animal*	
**Outcome measures**			
*Infarct size coefficient of variation*	Continuous	%	NA
*Mortality rate*	Continuous	%	NA

* Only categories represented by at least 5 control groups were included in the analysis to avoid statistically inadequate attribution of explanatory value to too small categories. Categories represented by less than 5 control groups were in the analysis included in an *Others* category. Further, some other reductions in number of categories were performed, as presented below.

** Elderly rats were defined as being >12 months of age at time of ischemic insult.

*** *Direct, mechanical* refers to all MCAo procedures where the MCA is mechanically occluded from the outside, for example by clips, cauterization or ligation.

**** Only methods including actions taken to ensure reperfusion (such as removing the occluding intraluminal filament or arterial clip) were considered transient.

Because many of the included articles/control groups lacked information about for example mortality rate, the researchers of those articles were contacted via e-mail with a gentle request to provide this information. In total, the authors of 310 articles were e-mailed, of which 183 (59%) complied.

**Figure 1:**
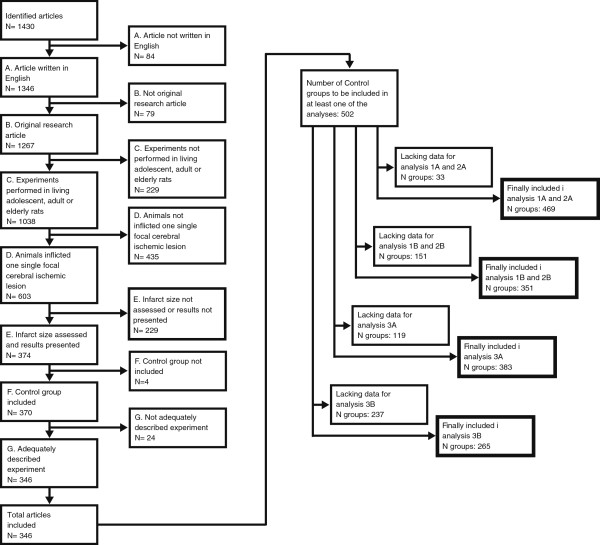
**When analyses were saturated, 1430 articles had been evaluated for inclusion in the meta-analysis.** After exclusion due to criteria A-G, 346 studies, describing 502 control groups, remained. Due to lack of certain pieces of information in some of the articles, not all 502 control groups could be included in all four multiple regression analyses, as shown in the thick-boarded boxes to the right in the figure.

Since power calculations for large multiple regression analyses are extremely complex to perform a priori, a saturation principle was adopted to determine a sufficient number of control groups to be included. After information from 300 control groups had been extracted, an interim analysis was performed, and then re-performed every 40–50 new control groups included. When the results had stabilized (no changes in overall trends, and only minor changes in p-values), no more articles were included. 502 control groups from 346 articles were finally included in the study [11,15-359] while 1084 articles were excluded (Figure 1).

### Processing of data

#### Category refinement

After extraction of data, categories represented by less than 5 control groups (corresponding to less than 1% of the material) were included in the *Others*-categories. This approach was motivated by the fact that these small categories otherwise would run the risk of being attributed high explanatory values that were not statistically substantiated, thus obscuring the influence of other categories.

In the *Other strain* category, the following variants were included: Long Evans rats, T-cell deficient nude rats, SHRSP, Fischer rats, Zucker rats, Hooded Wistar rats, Lewis rats, Holtzmann rats and Swiss albino rats.

The variable *Sex* finally contained four categories, since males formed the first category, females that were not explicitly ovariectomized were separated from ovariectomized females in a second and third category, and control groups using mixed or unspecified animals were grouped in a fourth category.

Fifteen various anesthesia regimens were reduced to four main categories and one *Others* category (in which for example methohexithal sodium, medetomedin and unspecified regimens were included). All inhalation anesthetics (isoflurane, halothane, sevoflurane, fluothane and enflurane) were included in the first category, while chloral hydrate was used frequently enough (and was not appreciably similar to any other category) to deserve a category of its own. The third category, *Ketamine*, included all ketamine containing regimens, such as ketamine/xylazine, ketamine/rumpun or ketamine only. Finally, all anesthetic regimens pertaining to the barbiturate or bensodiazepine groups, such as pentobarbital and diazepam, formed a fourth category.

The variable *Blood gases/O*_*2*_*saturation analyzed* was initially registered as the separate variables *Blood pH analyzed, Blood oxygen saturation analyzed* and *Blood carbon dioxide analyzed*, however these three were so highly correlated that they were thought better to be represented by only one variable.

Regarding the techniques for causing focal ischemic lesions, all intraluminal filament procedures were reduced to one single category. All direct occlusion techniques, based on craniectomy followed by physical occlusion of the MCA by means of a clip, suture, hook or cauterizer, formed the *Direct* category, while emboli techniques were clumped up in a third category. Photothrombotic procedures and methods of endothelin injection defined the fourth and fifth categories, respectively. It should however be noted that the occlusion time was accounted for in another variable and that the choice of different filaments were analyzed separately.

The filament categories, used for the analyses addressing hypotheses 3A and 3B, were also reduced. The uncoated filaments, a seemingly homogenous group, formed the first category, while silicone and resin coating were put in the *Silicone* category. Poly-L-Lysine formed a category of its own, while other rare coating techniques (including for example heparin coating, “glue coating” and paraffin coating) were put in a separate category together with unspecified coating techniques.

The procedures used for infarct evaluation were reduced to four categories. The most frequently used technique, 2,3,5-triphenyltetrazolium chloride staining, defined the first category, while radiologic methods (in the majority of cases magnetic resonance imaging, but in a few cases computed tomography) were put in a *Radiology* category. Various acidic/basic staining techniques (such as hematoxylin/eosin, cresyl violet and thionine) was, together with silver staining (used in only one of the included studies), included in category number three, while immunohistological methods were put in a fourth category.

Edema correction can be performed in different ways [360,361]. It was initially the intention to register not only if, but also which type of, edema correction was used. However, it soon turned out that this was not specified in a sufficient number of articles to perform a meaningful analysis. It was therefore only registered if edema correction had been used or not.

Concerning the exclusion procedures, the first category included all control groups in which no exclusion criteria were explicitly adopted. In the second category, control groups in which surveillance of blood flow reduction (for example using laser-doppler), with the plausible aim to exclude the absence of such, were put. The third, fourth and fifth categories contained control groups from articles in which lack of functional deficit, too small infarct size or other pathology (including intracerebral hemorrhage), respectively, were stated to be exclusion criteria. It should be noted that control groups from articles accounting for multiple exclusion criteria were registered in more than one of the exclusion categories.

#### Definition of continuous variables

*Animal exclusion rate* was defined as the percentage of rats excluded due to other reasons than mortality from induction of focal cerebral ischemia until the final infarct size assessment. *Time after focal ischemia for evaluation of damage* was defined as the time from cerebral blood flow obstruction until sacrifice. The outcome *Infarct size coefficient of variation* was defined as the standard deviation of the infarct volume divided by the average infarct volume. Irrespective of how the infarct size is presented; as percentage of the whole brain, as percentage of the hemisphere or in cubic millimeters, this calculation provides a strictly defined, and inter-comparable, measure of the infarct size variability. The other outcome, *Mortality*, was defined as the unintended mortality in the control group, from induction of focal cerebral ischemia until infarct size assessment, as a percentage of the whole group.

#### Statistical analyses

A priori, six main hypotheses (1A-3B) were put (as aforementioned):

1. Rat strain affects (A) infarct size variability and (B) mortality

2. Type of focal ischemia procedure affects (A) infarct size variability and (B) mortality.

3. In studies using the intraluminal filament method, the type of filament affects (A) infarct size variability and (B) mortality.

Obviously, several additional hypotheses could be tested in the information compiled from the studies, but the higher number of hypotheses, the higher the risk of finding falsely significant results due to multiple comparisons (type I errors). However, due to the risk of type II-errors, corrections for multiple comparisons were not performed, calling for separate assessment of the six hypotheses.

All category variables were dummy-converted before analysis (Table 1). For categorical variables with more than two categories, the most common category was chosen to be the reference category. For binary variables, it does not matter which one is made the reference, why [No] (in other words, the lack of a specific methodological ingredient) was consistently chosen. The data were subsequently analyzed using multiple regression analyses with backward variable exclusion. This step identified which factors significantly affected the outcomes *Infarct size coefficient of variation* and *Mortality*, respectively. Next, an enter model, in which the variables from the backward procedure were complemented by lacking dummy variables, was performed (the enter models with the variables found significant in the backward analyses are presented in Tables 2, 3, 4, 5). The analyses were weighted according to the number of animals used in each group; hence, a study including 5 animals in the control group was given less impact than a study including 20 animals. In total, four large multiple regression models (one for hypotheses 1A and 2A, one for hypotheses 1B and 2B, one for hypothesis 3A and one for hypothesis 3B) were set up, testing the combined effects of all available factors on the respective outcome measure (*Infarct size coefficient of variation* or *Mortality rate*). Hence, all models controlled for the factors listed in Table 1 when testing the stated hypotheses. All statistical calculations were performed in SPSS (Version 20, IBM Corporation, Armonk, NY, USA). P-values <0.05 were considered statistically significant. Data were presented as mean ± standard deviation or, when presenting results from the meta-analysis, with 0.95 confidence interval shown within brackets. It should be noted that the percent changes (regression coefficients) in *Infarct size coefficient of variation* and *Mortality rate* are presented in absolute, not relative, terms. In other words, if a certain variable decreases *Mortality rate* with 10%, it means that the mortality would decrease from for example 40% to 30%, and not merely from 40% to 36%.

**Table 2:** Regression formula for hypotheses 1A and 2A

**Regression formula for the effect of Strain and Type of middle cerebral artery occlusion procedure on Infarct size coefficient of variation (hypotheses 1A and 2A)**					
**Variable (reference category)**	**Variable categories**	**Regression coefficient**	**0.95 confidence interval for regression coefficient**		**p-value**
Constant	NA	23.1	8.3	38.0	0.002
*Strain (Sprague Dawley)*	*Wistar*	−6.2	−11.5	−0.9	0.023
	*SHR*	−1.7	−11.0	7.5	0.710
	*WKY*	19.0	−0.8	38.8	0.059
	*Other Strains*	20.7	10.7	30.8	0.000
*Type of middle cerebral artery occlusion procedure (Intraluminal filament)*	*Direct, mechanical*	4.2	−3.4	11.8	0.274
	*Embolic*	14.7	3.4	26.0	0.011
	*Photothrombotic*	10.1	−5.2	25.5	0.196
	*Endothelin injection*	23.1	9.3	36.9	0.001
*Sex (Male)*	*Female*	−0.6	−19.7	18.5	0.951
	*Ovx female*	2.3	−16.7	21.4	0.810
	*Mix or not specified*	−11.5	−21.5	−1.5	0.024
*Elderly rats*	[Yes]	−23.6	−39.6	−7.7	0.004
*Weight*	[Continuous; Grams]	0.1	0.0	0.1	0.001
*Type of anesthetic (Inhalation anesthetic)*	*Chloral Hydrate*	−11.5	−16.9	−6.1	0.000
	*Ketamine*	2.9	−4.4	10.2	0.430
	*Barbiturates and bensodiazepines*	−8.4	−17.6	0.9	0.076
	*Other anesthetic*	−13.6	−22.6	−4.6	0.003
*Awakening of rats during occlusion (No)*	[Yes]	8.8	0.9	16.7	0.028
*Temperature feedback system (No)*	[Yes]	−6.9	−12.4	−1.5	0.013
*Blood hemoglobin concentration analyzed (No)*	[Yes]	10.9	3.0	18.8	0.007
*Occlusion duration (Short transient)*	*Long transient*	1.9	−3.9	7.8	0.519
	*Permanent*	−16.6	−24.1	−9.1	0.000
*Time after focal ischemia for evaluation of damage*	[Continuous; Hours]	0.0051	0.0005	0.0096	0.030
*Criteria for excluding rats (None)*	*Observed absence of cerebral blood flow reduction*	−7.7	−12.4	−2.9	0.002
	*Lack of functional deficit*	−2.9	−8.6	2.7	0.310
	*Too small infarct*	0.1	−9.6	9.9	0.981
	*Other pathology in animal*	18.6	12.7	24.6	0.000

Variables excluded by statistical software due to too low explanatory value: Intubation, Postoperative antibiotics, Blood pressure monitoring, Heart rate monitoring, Blood gases/O2 saturation analyzed, Blood glucose concentration analyzed, Type of infarct size evaluation, Edema correction used, Blinding of infarct size determination procedure.

**Table 3:** Regression formula for hypotheses 1B and 2B

**Regression formula for the effect of Strain and Type of middle cerebral artery occlusion procedure on Mortality rate (hypotheses 1B and 2B)**					
**Variable (reference category)**	**Variable categories**	**Regression coefficient**	**0.95 confidence interval for regression coefficient**		**p-value**
Constant	NA	17.1	13.9	20.3	0.000
*Strain (Sprague Dawley)*	*Wistar*	1.0	−2.4	4.4	0.551
	*SHR*	−6.9	−12.8	-.87	0.025
	*WKY*	−8.3	−19.3	2.8	0.141
	*Other Strains*	4.4	−1.1	9.9	0.113
*Type of middle cerebral artery occlusion procedure (Intraluminal filament)*	*Direct, mechanical*	−10.7	−15.1	−6.2	0.000
	*Embolic*	12.1	6.9	17.3	0.000
	*Photothrombotic*	−5.6	−15.3	4.2	0.262
	*Endothelin injection*	−9.7	−16.8	−2.6	0.007
*Type of anesthetic (Inhalation anesthetic)*	*Chloral Hydrate*	4.1	0.17	8.1	0.041
	*Ketamine*	3.2	−1.1	7.4	0.144
	*Barbiturates and bensodiazepines*	1.1	−4.6	6.8	0.710
	*Other anesthetic*	3.0	−1.9	8.0	0.230
*Awakening of rats during occlusion (No)*	[Yes]	9.1	3.9	14.2	0.001
*Heart rate monitored (No)*	[Yes]	−3.3	−7.5	0.94	0.127
*Blood glucose concentration analyzed (No)*	[Yes]	−2.8	−6.1	0.53	0.099
*Type of infarct size evaluation (TTC)*	*Radiology*	5.4	−0.11	10.9	0.055
	*Acidic/Basic stain or silver stain histology*	−1.2	−4.7	2.4	0.526
	*Immunohistology*	10.4	3.2	17.6	0.005
*Criteria for excluding rats (None)*	*Observed absence of cerebral blood flow reduction*	−4.0	−6.9	−1.1	0.008
	*Lack of functional deficit*	0.15	−3.4	3.7	0.933
	*Too small infarct*	0.22	−5.1	5.6	0.935
	*Other pathology in animal*	−3.4	−7.1	0.40	0.079

Variables excluded by statistical software due to too low explanatory value: Sex, Elderly rats, Weight, Intubation, Temperature feedback system, Blood pressure monitored, Blood gases/O_2_ saturation analyzed, Blood hemoglobin concentration analyzed, Postoperative antibiotics, Occlusion duration, Time after focal ischemia for evaluation of damage, Edema correction used, Blinding of infarct size determination procedure.

**Table 4:** Regression formula for hypothesis 3A

**Regression formula for the effect of Occluding filament type on Infarct size coefficient of variation (hypothesis 3A)**					
**Variable (reference category)**	**Variable categories**	**Regression coefficient**	**0.95 confidence interval for regression coefficient**		**p-value**
Constant	NA	49.1	38.7	59.4	0.000
*Occluding filament type (Uncoated)*	*Silicon coated*	−12.7	−18.3	−7.0	0.000
	*Poly-L-Lysine coated*	3.1	−4.8	11.0	0.444
	*Other coatings*	3.1	−5.0	11.3	0.451
*Strain (Sprague Dawley)*	*Wistar*	−2.1	−8.3	4.1	0.507
	*SHR*	−3.0	−17.2	11.2	0.679
	*WKY*	16.1	−5.6	37.8	0.146
	*Other Strains*	25.9	11.0	40.7	0.001
*Sex (Male)*	*Female*	3.3	−17.0	23.7	0.747
	*Ovx female*	12.1	−8.5	32.6	0.248
	*Mix or not specified*	−5.0	−15.1	5.2	0.335
*Elderly rats*	[Yes]	−17.8	−38.4	2.9	0.091
*Type of anesthetic (inhalation anesthetic)*	*Chloral Hydrate*	−12.0	−18.7	−5.4	0.000
	*Ketamine*	4.5	−4.3	13.2	0.315
	*Barbiturates and bensodiazepines*	−12.4	−24.8	0.00	0.050
	*Other anesthetic*	−14.2	−24.1	−4.4	0.005
*Awakening of rats during occlusion (No)*	[Yes]	10.4	2.1	18.6	0.014
*Temperature feedback system (No)*	[Yes]	−7.6	−14.1	−1.2	0.021
*Blood pressure monitored (No)*	[Yes]	−12.0	−21.4	−2.6	0.013
*Heart rate monitored (No)*	[Yes]	14.9	5.4	24.4	0.002
*Blood gases/O*_*2*_*saturation analyzed (No)*	[Yes]	11.5	1.8	21.1	0.021
*Blood hemoglobin concentration analyzed (No)*	[Yes]	10.7	1.5	19.9	0.023
*Occlusion duration (Short transient)*	*Long transient*	−3.3	−9.3	2.8	0.288
	*Permanent*	−21.4	−30.1	−12.8	0.000
*Time after focal ischemia for evaluation of damage*	[Continuous; Hours]	0.000036	−0.000013	0.000087	0.157
*Blinding of infarct size determination procedure (No)*	[Yes]	−3.8	−9.9	2.2	0.211
*Criteria for excluding rats (None)*	*Observed absence of cerebral blood flow reduction*	−7.4	−13.1	−1.7	0.011
	*Lack of functional deficit*	−5.6	−12.0	0.7	0.083
	*Too small infarct*	7.6	−4.5	19.6	0.219
	*Other pathology in animal*	16.2	8.7	23.6	0.000

Variables excluded by statistical software due to too low explanatory value: Weight, Intubation, Postoperative antibiotics, Blood glucose concentration analyzed, Type of infarct size evaluation, Edema correction used.

**Table 5:** Regression formula for hypotheses 3B

**Regression formula for the effect of Occluding filament type on Mortality rate (hypothesis 3B)**					
**Variable (reference category)**	**Variable categories**	**Regression coefficient**	**0.95 confidence interval for regression coefficient**		**p-value**
Constant	NA	16.8	13.2	20.4	0.000
*Occluding filament type (Uncoated)*	*Silicon coated*	−1.2	−4.7	2.4	0.516
	*Poly-L-Lysine coated*	3.0	−1.3	7.4	0.171
	*Other coatings*	−3.6	−9.0	1.8	0.188
*Type of anesthetic (Inhalation anesthetic)*	*Chloral Hydrate*	5.0	0.57	9.4	0.027
	*Ketamine*	3.1	−1.6	7.8	0.198
	*Barbiturates and bensodiazepines*	6.6	−0.91	14.1	0.085
	*Other anesthetic*	5.6	−0.04	11.2	0.052
*Awakening of rats during occlusion (No)*	[Yes]	9.7	4.5	14.8	0.000
*Heart rate monitoring (No)*	[Yes]	−6.5	−10.8	−2.2	0.003
*Type of infarct size evaluation (TTC)*	*Radiology*	13.3	6.5	20.1	0.000
	*Acidic/Basic stain or silver stain histology*	−1.2	−5.2	2.9	0.576
	*Immunohistology*	10.8	1.6	20.0	0.022
*Criteria for excluding rats (None)*	*Observed absence of cerebral blood flow reduction*	−5.5	−8.6	−2.4	0.001
	*Lack of functional deficit*	.39	−3.5	4.2	0.843
	*Too small infarct*	−7.4	−14.2	-.56	0.034
	*Other pathology in animal*	−1.2	−5.3	2.9	0.577

Variables excluded by statistical software due to too low explanatory value: Strain, Sex, Elderly rats, Weight, Intubation, Temperature feedback system, Blood pressure monitoring, Blood gases/O_2_ saturation analyzed, Blood hemoglobin concentration analyzed, Blood glucose concentration analyzed, Postoperative antibiotics, Occlusion duration, Time after focal ischemia for evaluation of damage, Edema correction used, Blinding of infarct size determination procedure.

### Protocol violations

It was originally planned to include the variable *Exclusion rate* to control for this confounder; however, too few articles presented the needed information. Even after all persistent e-mail correspondence, such a high number of studies lacked this variable that including it would have seriously hampered the analyses’ power. The variable was therefore omitted.

*Electroencephalographic surveillance* was only utilized in one of the included studies, and this variable could thus not be analyzed. It was therefore omitted.

## Results

### Impact of rat strain on infarct size coefficient of variation and mortality: Hypotheses 1A-B

**Figure 2:**
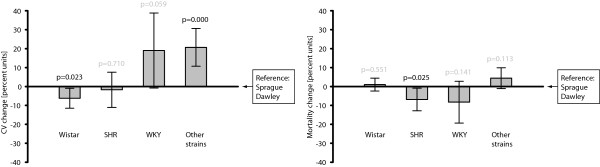
**The choice of strain significantly affected the *Infarct size coefficient of variation, *so that the *Wistar *rendered lower variability than *Sprague Dawley, *which was chosen as the reference category.** The *Other strains* category increased variability in comparison to *Sprague Dawley*. Regarding mortality rate, the effects of animal strain was limited to a slight decrease from using *SHR*. N = 469 and 351, respectively, in the two analyses/graphs. The bars represent 0.95 confidence intervals.

*Strain* significantly affected both *Infarct size coefficient of variation* and *Mortality rate*. *Wistar* had the strongest negative regression coefficient, and rendered significantly lower variability (−6.2%, 0.95 CI: -11.5 to −0.9%, p = 0.023) than the well-used *Sprague Dawley*, while the category *Other strains* had significantly higher variability (+20.7%, 0.95 CI: +10.7 to +30.8%; p = 0.000; Figure 2; Table 2).

The only effect of *Strain* on *Mortality rate* was that *SHR* seemed to render lower percentages (−6.9%, 0.95 CI: -12.8 to −0.87%; p = 0.025; Figure 2; Table 3).

The multiple regression analysis addressing hypotheses 1A and 2A included 469 control groups, while the analysis for hypotheses 1B and 2B included 351 control groups (Figure 1). These regression formulae had r^2^ of 0.34 and 0.31, respectively, meaning that they explained 34% and 31% of the variation in the outcomes *Infarct size coefficient of variation* and *Mortality rate*.

### Impact of focal ischemia procedure on infarct size coefficient of variation and mortality: Hypotheses 2A-B

**Figure 3:**
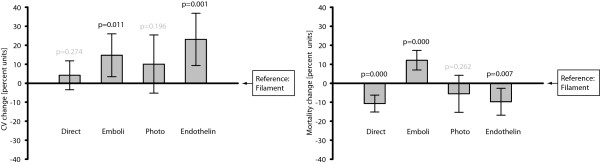
**Concerning *Infarct size coefficient of variation, *the general trend was that the intraluminal filament procedure, here chosen to be the reference category, resulted in lower percentages than did the other methods.** The emboli and endothelin injection methods rendered significantly higher variability. *Mortality rate* was clearly influenced by choice of induction procedure, with higher percentages in the emboli studies, while the direct and endothelin procedures had decreased numbers of deaths in comparison to the intraluminal filament method. N = 469 and 351, respectively, in the two analyses/graphs. The bars represent 0.95 confidence intervals.

Regarding *Infarct size coefficient of variation*, all analyzed surgical procedures had positive regression coefficients, indicating higher variability than in the intraluminal filament method, here chosen to be the reference. This trend was significant for the *Emboli* (+14.7%, 0.95 CI: +3.4 to +26.0%; p = 0.011) and *Endothelin* (+23.1%, 0.95 CI: +9.3 to +36.9%; p = 0.001) categories (Figure 3, Table 2).

The emboli (+12.1%, 0.95 CI: +6.9 to +17.3%; p = 0.000) method rendered higher mortality than the intraluminal filament method, while the direct (−10.7%, 0.95 CI: -15.1 to −6.2%; p = 0.000) and endothelin (−9.7%, 0.95 CI: -16.8 to −2.6%; p = 0.000) methods resulted in lower mortality (Figure 3, Table 3).

### Impact of type of filament on infarct size coefficient of variation and mortality: Hypotheses 3A-B

**Figure 4:**
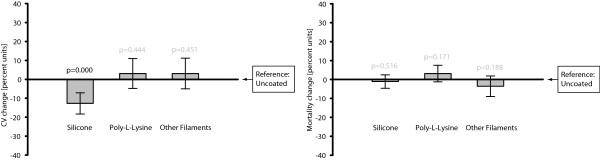
**Silicone coated filaments rendered lower infarct size coefficient of variation than the uncoated filaments.** Poly-L-Lysine coated and other filaments resulted in infarct size coefficients of variation comparable to the uncoated counterparts. No effect on *Mortality rate* from the choice of intraluminal filament type was seen. N = 383 and 265, respectively, in the two analyses/graphs. The bars represent 0.95 confidence intervals.

In studies in which the intraluminal filament method had been used, silicone coating of the occluding filament substantially and significantly lowered *Infarct size coefficient of variation* compared to uncoated filaments (−12.7%, 0.95 CI: -18.3 to −7.0%; 0.000). It should also be noted that *Poly-L-Lysine* had a positive regression coefficient, indicating a slight trend of increased rather than decreased variability in comparison with the reference category (Figure 4, Table 4).

The choice of filament coating had no significant effects on mortality, and regression coefficients were generally small (Figure 4, Table 5).

The multiple regression analyses addressing hypotheses 3A and 3B included 383 and 265 control groups, respectively, all using the intraluminal filament technique (Figure 1). These regression formulae had r^2^ of 0.40 and 0.27.

### Background data

In the 502 control groups finally included, the *Infarct size coefficient of variation* were on average 28.9 ± 21.3%, with a range from 1.7 to 148%. *Mortality rate*, the other outcome variable, averaged 15.1 ± 13.5%, ranging from 0 to 60.4%.

**Figure 5:**
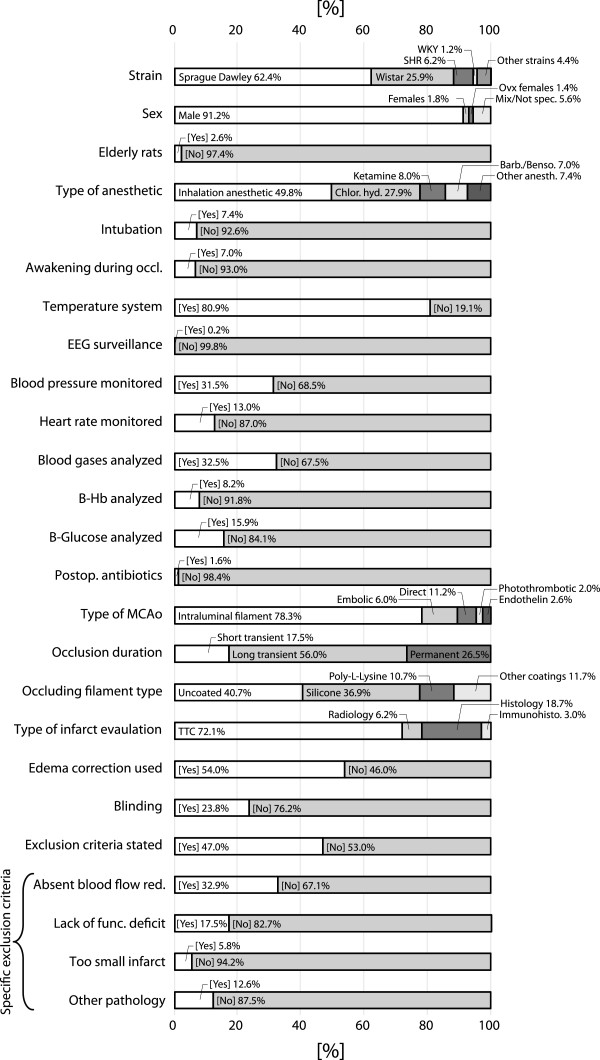
**Frequencies of registered categories in the 502 included control groups.** The specific exclusion criteria are presented separately in the last 4 bars. Many of the variable names are abbreviated in the figure; for extended description, see Table 1. “Histology” in the bar “Type of infarct evaluation” refers to acidic/basic stain or silver stain histology. EEG = Electroencephalography, B = Blood, Hb = Hemoglobin.

The average number of animals in the 502 control groups, which was used for weighing the studies’ impact in the analyses, was 9.0 ± 7.7, with a range from 3 to 145. Mean rat body weight in the included studies was 294.9 ± 61.0 g, ranging from group means of 190 to 779 g. Cerebral damage was evaluated on average 165.5 ± 506.3 hours after ischemic insult, but this data was heavily skewed, with a median of 24 hours. The exclusion rate (due to other reasons than mortality) averaged 8.9 ± 9.8% in the few studies in which this information was available. Frequencies of the different classifications in the registered categorical variables are presented in Figure 5.

## Discussion

The most important findings in the current hypothesis-driven meta-analysis was that the Wistar strain and intraluminal filament procedure using a silicone coated filament resulted in smallest infarct size variability. The direct and endothelin methods rendered the lowest mortality rates, while the emboli method increased mortality when compared to the intraluminal filament method. A number of interesting observations regarding the control variables were also made, such as the significant impact of the exclusion criterion *Observed absence of cerebral blood flow reduction* on variability (Table 2) and the effect of awakening the rats during occlusion on mortality (Table 3). However, since these accidental findings were not part of the original hypothesis, we refrain from drawing any conclusions about them, and refer to Tables 2, 3, 4, 5 for the interested reader.

The high infarct size variability in rodent focal ischemic models is a problem that burdens the entire experimental stroke field, and has been commented in several reviews [2,362]. The problem with high outcome variability is that a higher number of animals is needed to get an adequate statistical power, which is problematic from both an ethical and economical point of view. The pressure from ethical boards on the researcher to minimize the number of animals used may be the main reason that the power of stroke experiments is often low. In the current meta-analysis, the average infarct size coefficient of variation was 28.9%, while the average number of animals included in the control groups were 9.0. If we assume that the animals in the included studies often are equally distributed between the treatment groups and control groups, these numbers can provide an estimate of the average statistical power in the studies. Given the abovementioned numbers and an alpha of 0.05, the chance of detecting a 20% difference between the groups would be merely 54.6% (if non-parametric tests are used instead or if more than two groups are included in the comparison, the power would be even lower). Under these circumstances, a negative result is marginally more interesting that tossing a coin. The use of low power designs risks serious publication bias which also makes meta-analyses of experimental stroke studies difficult to interpret properly, since there is probably an unknown number of unpublished studies that cannot be weighed in. In addition to using means to decrease variability, it is of fundamental importance to use a sufficient number of animals/replicates to render an acceptably high power.

Mortality can be another confounding factor in experimental stroke research, at least if it is not reported in the article. With parametric statistical methods, incorporating mortality in for example the infarct size calculations is not uncomplicated from a statistical point of view, which is probably why the mortality is often simply not mentioned. Non-parametric models may offer an alternative approach [363], but irrespective of how the main outcome is statistically assessed, the importance of reporting mortality and other exclusion criteria cannot be over-emphasized. For example, if mortality rate is omitted, a substance that kills all rats with large infarcts may seem to decrease infarct sizes, since only the animals with small infarcts will survive in the treatment group. Unfortunately, mortality rate is usually not reported in experimental stroke studies. In fact, for only 35.3% of the included control groups an account of unintended deaths was provided; and this was the most frequently requested item in our e-mail correspondence.

A few previous studies have assessed the influence of the rat strain on experimental stroke outcomes, however with conflicting results. Spontaneously hypertensive rats (SHR) have, probably because of the implications of hypertension in stroke pathophysiology, attracted some attention. Since this strain often sustains larger infarcts [364], the infarct size coefficient of variation has in a few studies been shown to be relatively low [8,365,366]. Others have argued that the use of another inbred strain – Fischer-344 rats – give the most consistent results [7,367,368], however this type may because of its variable vascular anatomy be unsuitable for the well-used intraluminal filament model [369]. Long Evans rats have also been proponed as a good model animal, because of the relatively consistent decrease in cerebral blood flow after intraluminal filament MCAo [370]. Many other studies have investigated differences between rat strains, however not focusing on variability, but rather on infarct sizes per se [371-373]. Except for the study emphasizing the unsuitability of Fischer-344 for intraluminal filament MCAo [369], we are only aware of one study aiming to compare mortality between strains. In this study, an intraluminal filament model rendered higher mortality in Fischer-344 rats than in Wistar and Sprague–Dawley rats [7]. It is not easy to summarize the conclusions in the existing literature, since the mentioned experiments have been performed under such different circumstances, but it seems that SHR might be attractive because of their low variability. In the current meta-analysis, there was a slight trend towards lower variability in the SHR strain compared to the reference category Sprague–Dawley, which however was far from reaching statistical significance. Regarding Long Evans and Fischer-344, these strains were used too rarely to be analyzed separately. As abovementioned, the strain that we found to render the lowest variability was the relatively well-used Wistar strain.

Very few studies have compared different methods of inducing focal cerebral ischemia. This is perhaps not surprising given that the effort of introducing an entirely new MCAo method in a laboratory is large enough to make many researchers reluctant to switch once a technique has been mastered. This lack of relevant studies underscores the importance of a meta-analysis as the current one. However, in a study by Gerriets et al. [10], an embolization technique was compared to an intraluminal filament procedure. The take-home message from that article was that even though infarct variability tended to be higher from the emboli method, as corroborated by the current study, it did not affect body temperature to the same extent as the filament method did. In another article, the use of microsurgical direct occlusion was advocated over the intraluminal filament method because the latter was thought to not only compromise blood flow to the MCA territory, but rather a larger part of the ICA territory [374].

Different types of filaments for the intraluminal method are much easier to compare, and have been assessed in several studies. Most of these studies have argued that silicone coated rather than uncoated or poly-L-lysine coated filaments should be used, because of a more consistent blood flow reduction [375], lower incidence of sub-arachnoid hemorrhage [14,376], higher success rate [376-378], lower mortality [377] and lower variability [368,376], even if arguments based on low variability also have been used to encourage the use of poly-L-lysine coating [379]. In the current analysis, it was found that silicone coated filaments are superior in terms of reducing infarct size variability, while no effects on mortality were found. Another study compared different brands of blunted nylon filaments, and found Ethilon to be superior to Nitcho [380].

### Strengths and weaknesses

The main strength of the current study is that it, based on hundreds of published studies, provides a composite understanding of how different methodological factors interact to affect outcome variability and mortality. However, since this method-investigating meta-analytical approach is relatively novel, we consider it important to highlight and discuss some aspects of the design:

• A multiple regression analysis assumes that the variables are linearly related, which evidently is not always true. For example, the effect of average body weight on variability could theoretically be U-shaped, with higher variability in young, not fully developed, rats and very old animals, than in adult animals. This is an inherent drawback, but multiple regression analysis still seemed the most attractive statistical method for the current purpose.

• There is a problem in investigating coefficients of variation in published studies, and weighing the impact of the included control groups by number of animals, since important sources of bias come into play. Researchers that know that their model render large variability will compensate by including more animals, thus giving more weight in the meta-analysis to studies with larger variability. We however believe that weighing the analysis by number of animals is the fairest alternative. Another problem is publication bias, since the studies rendering the largest coefficients of variation probably to large part remain unpublished, and cannot be assessed.

• Even if this meta-analysis controls for many confounders by its broad approach, there is complexity and heterogeneity in the underlying experiments that is far beyond our reach. For example, the impact of different rat vendors [381], the skill of the surgeon and the suitability of using specific rat strains for certain surgical procedures are not accounted for. For mathematical reasons, categories have also, as stated in the Materials and Methods section, been reduced to larger categories, meaning that differences within categories may be lost. There are for example numerous variations within the different MCAo techniques, and it is likely that the best embolization procedure renders a lower variability than the worst intraluminal filament paradigm, even if the last-mentioned method proved superior on a general level.

• The meta-analysis includes 502 control groups from only 346 published reports, meaning that several studies described more than one control group. We have in the statistical analysis regarded control groups from the same study as independent, which is not statistically stringent. However, if categories had been created for all separate studies, the entire analysis would have been impossible to perform, and thus this imperfection is an inevitable problem with the chosen approach.

## Conclusions

The choice of methodological parameters, such as rat strain and infarct surgical procedures, is of utmost importance for consistent and reliable results. As found in the meta-analysis, the effect sizes were large, with many parameters by themselves increasing or decreasing variability and mortality with more than 10% (in absolute terms).

Finally, it deserves to be emphasized that this analysis does not encompass all perspectives on the suitability of focal stroke models. Even if infarct size coefficient of variation and mortality are important components, other aspects, not least similarity of the model to the clinical situation, emphasizing the importance of the embolic model [382], must be taken into consideration when planning experiments.

## Abbreviations

MCAo: Middle cerebral artery occlusion; MCA: Middle cerebral artery; SHR: Spontaneously hypertensive rat; WKY: Wistar Kyoto rats; Ovx: Ovariectomized; CV: Coefficient of variation, calculated as [standard deviation/average]; CI: Confidence interval; EEG: Electroencephalography; Hb: Hemoglobin.

## Competing interests

The authors declared that they have no competing interests.

## Authors’ contributions

JOS contributed to designing the study, extracted data, performed the outcome analyses and drafted the manuscript. EI contributed to designing the study, extracted data and revised the manuscript. AT and ET contributed to designing the study and revised the manuscript. All authors read and approved the final manuscript version before submission.
